# Robot-assisted pedicle screw placement by using optical navigation

**DOI:** 10.3389/frobt.2026.1774473

**Published:** 2026-04-15

**Authors:** Ayoob Davoodi, Ruixuan Li, Aidana Massalimova, Fabio Carrillo, Gianni Borghesan, Christoph J. Laux, Philipp Fürnstahl, Kathleen Denis, Emmanuel Vander Poorten

**Affiliations:** 1 Department of Mechanical Engineering, Robot-Assisted Surgery Group, KU Leuven, Leuven, Belgium; 2 Research in Orthopedic Computer Science, University Hospital Balgrist, University of Zurich, Zürich, Switzerland; 3 BioMechanics (BMe), Smart Instrumentation Group, KU Leuven, Leuven, Belgium

**Keywords:** optical navigation, orthopedic surgery, pedicle screw placement, robot-assisted drilling, spine surgery navigation

## Abstract

Pedicle screw placement is the gold standard for spinal pathologies that require surgical stabilization. Accurate screw placement is important to avoid severe complications, but ensuring accuracy remains a challenge. This work proposes an optical camera-based robot-assisted drilling system for spine surgery. Preoperative CT scans are used to plan screw trajectories in both synthetic phantoms and *ex-vivo* ovine spines. After robotic drilling, postoperative scans are registered to the preoperative plan to quantify drilling accuracy. In total, 48 screws are drilled into *ex-vivo* ovine spines, producing a median entry point distance error of 1.51 mm (IQR 0.91mm, 2.10 mm) and an angle error of 2.30° (IQR 1.28° , 3.13° ), where 95.83% of the placements were clinically successful. Optical camera-based robot-assisted drilling system for spine surgery may increase accuracy by providing precise spatial registration between the patient anatomy and the robotic system, enabling accurate alignment and execution of the drilling task.

## 1 Introduction

Pedicle screw placement (PSP) consists of implanting a plurality of screws into vertebral pedicles for spinal stabilization (Ledonio et al., 2013). Considering the proximity of the surgical field Considering the proximity of the surgical field to the main blood vessels and the central nervous system, consistent accuracy could help reduce complications and shorten recovery time after surgery (Song et al., 2022).

Currently, computed tomography (CT)-based navigation and fluoroscopy-based navigation are the most popular computer-assisted spine navigation systems (Hussain et al., 2020; Karkenny et al., 2019). These systems have advanced significantly over the years in terms of screw placement accuracy. They offer advantages such as shorter surgical times and improved surgical outcomes, but also present disadvantages such as increased radiation exposure (Farshad et al., 2017; Fatima et al., 2021). Gelalis et al. reported success rates ranging from 89% to 100% using CT-based navigation and from 81% to 92% using fluoroscopy-based navigation (Gelalis et al., 2012). However, these image-guided modalities increase radiation exposure and may interrupt the surgical workflow (Klingler, 2021). Additionally, the limited field of view of fluoroscopy-based navigation systems affects their application (Li S. et al., 2020). Recent advances include the integration of real-time navigation technologies, such as optical navigation and augmented reality that do not depend on radiation (Yamout et al., 2023). Although the precision of computer-assisted screw placement has significantly improved (Matur et al., 2023), the pursuit of improved clinical outcomes has led to the introduction of robotics in conjunction with computer-assisted navigation. The enhanced precision and repeatability offered by robotics form the main motivation for including them. The state-of-the-art in this field includes optical tracking systems (OTS) and robot-assisted PSP systems.

### 1.1 Robot-assisted PSP

Li et al. performed a meta-analysis showing that a robot-assisted fluoroscopy-based system achieves 88.9% grade A screws according to the Gertzbein-Robbins (GR) classification Robbins and Gertzbein (1989), compared to 84.0% with fluoroscopy-based free-hand screw placement Li H.-M. et al. (2020). The learning curve for the robot-assisted PSP technique was significantly fast for new surgeons who worked with robotic navigation for the first time (Kim et al., 2017; Urakov et al., 2017). For instance, Kim et al. investigated the learning curve in their early 8 and later 29 cases. They found a decrease in screw placement time from 14.86 to 9.03 min and a decrease in fluoroscopy capturing time from 27.5 to 18.5 s. The robot-assisted screw insertion has been shown to reduce intraoperative blood loss and length of hospital stay (Asada et al., 2024). Several commercial robotic systems have been utilized in spine surgery, such as the Mazor X (Medtronic Navigation, Louisville, CO, USA) and the ExcelsiusGPS (Globus Medical, Inc., Audubon, PA, USA). These systems are programmed to assist surgeons by guiding predetermined drilling trajectories during surgery. A fiducial marker is temporarily attached to the robot arm and registered with intraoperative scans (e.g., O-arm by Medtronic). Subsequently, the robot arm is positioned within the O-arm’s field of view and aligned with the planned trajectory. Connor et al. placed 90 pedicle screws on patients using the Mazor X Stealth Edition robot and reported a 100% Grade A success rate using the GR classification (O’Connor et al., 2021). Another robotic system, TiRobot (TINAVI Medical Technologies, China), provides a guide holder mounted on the robot arm that automatically moves to the planned trajectory according to the surgical plan, registered with C-arm (Han et al., 2019). Surgeons can then drill guide pins and place screws through this holder. The clinically acceptable accuracy of this robotic-assisted screw placement was 98.7% compared to the free-hand group 92.2% (Chen et al., 2020).

Previous robotic systems have been limited to providing passive drill guides, requiring manual manipulation by the surgeon. Instead, compared to manual drilling, autonomous robotic drilling could enhance precision, respond faster upon disturbances or breaches and hence potentially improve surgical outcomes. It is thus of interest to develop and explore the use of robotic arms that respond autonomously offering a functionality beyond today’s commercial robotic arms that remain limited to merely holding a passive drill guide.

### 1.2 Optical tracking navigation

OTS have been previously used in various orthopedic interventions. Their primary function was to track tools. Often, these systems were combined with other navigation systems, such as CT and fluoroscopy (Hsia et al., 2024; Li T. et al., 2023; Luo and Badreddin, 2022). Robotic systems supported by OTS have facilitated guiding instruments along planned trajectories in procedures like total knee arthroplasty (Batailler et al., 2021; Shatrov et al., 2021; Siddiqi et al., 2021) and total hip replacement surgeries (Luo et al., 2020; Hu et al., 2021). BrainLab’s Curve (Brainlab AG, Munich, Germany) that uses intraoperative CT La Rocca et al. (2023), is arguably one of the most widely used commercial optical navigation systems for PSP. While a great support during current interventions also this system relies on the surgeon’s experience to execute difficult gestures with extreme precision. Recent advancements in optical navigation aim to optimize the field of view by attaching the optical camera to the end effector of robot, allowing movement of the camera and reducing the occurrence of occlusion of the tool maker (Han et al., 2023b; Han et al., 2023a).

### 1.3 Motivations and contributions

However, prior robotic PSP systems only cover a part of the procedure. The robot merely provides a programmable guide letting the surgeon to perform the drilling procedure manually. Due to the inherent flexibility of the spine, the interaction forces could bend the spine affecting the accuracy. The human’s response time is also lower than that of a robot. If a breach would occur the slow response could thus be more damaging. To avoid the effects of radiation during fluoroscopy-based procedures, this work explores intraoperative guidance solely based on OTS. OTS could potentially improve intraoperative precision by allowing continuous accounting for variations in the positions of the patient and drilling tool.

Inaccurate tool calibration is an error source in optical navigation. In orthopedic applications such as PSP, deviations in tool geometry or mounting may arise due to equipment sterilization, exchange or wear of drill bits, or the deterioration or shift of optical markers over time. As a result, it is necessary to regularly update and verify system calibration to ensure that actual tool calibration is sufficiently accurate.

Since it is important not to further lengthen the procedural time, a fast and reliable calibration procedure is vital. This method should be further easy to execute so that operators with limited expertise in robotics could also make use of it.

As a step towards a practical intraoperative, radiation-free optical-guided robot-assisted PSP, the main contributions of this paper are: (a) a fully integrated robotic drilling framework for PSP that unifies the robot, drilling tool, and optical tracking system via multi-stage spatial calibration to ensure precise alignment with the preoperative trajectory; (b) practical calibration methods that are compatible with operating room requirements and ensure accurate tool positioning; (c) a hybrid position-force control scheme to control the drill-vertebrae interaction force and lower the impact of unintended disturbances; (d) comprehensive experimental evaluation of robotic drilling performance on synthetic phantoms and *ex-vivo* ovine spines, demonstrating consistent geometric accuracy across complex vertebral anatomies.

This paper is organized as follows: Section 2 describes the method. Section 3 reports the experimental results. Section 4 discusses the proposed system and offers a conclusion at the end.

## 2 Materials and methods

### 2.1 System overview

**FIGURE 1:**
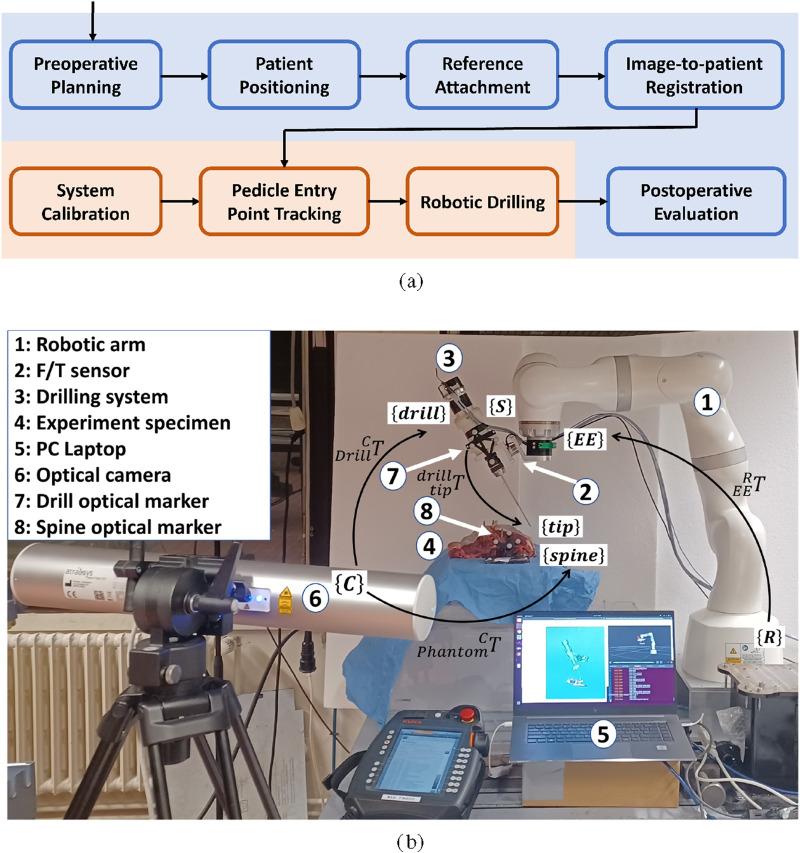
**(a)** Overview of the proposed clinical workflow integrating optical navigation with robotic drilling of this study. **(b)** Illustration of the experimental setup with a custom-designed drilling system.

Figure 1a summarises the envisioned clinical workflow integrating the developed robotic system. The blue blocks represent standard clinical PSP steps, while the orange block indicates the additional robotic component introduced in this work. A preoperative CT scan is first acquired. The vertebrae are segmented and screw trajectories are planned within the centre of each pedicle with adequate cortical clearance. In the operating theatre, the patient is positioned prone and a rigid optical reference marker is attached to a spinous process or the iliac crest. The preoperative CT is registered to the patient using fiducial-based or surface-based registration, establishing the transformation between image and patient space. In conventional robotic PSP systems, the robot positions a passive drill guide and the surgeon performs drilling manually. In contrast, the proposed workflow enables autonomous drilling. The drill-tip calibration described in Section 2.2 is performed, typically prior to surgery. During surgery, the optical tracking system continuously measures both the patient and drill markers. The robot aligns the drill axis with the planned trajectory while compensating for relative patient motion as described in Section 2.3. Drilling is then executed under hybrid position–force control (Section 2.4) to maintain alignment and regulate interaction forces. Postoperative evaluation follows standard PSP assessment procedures.

The experimental setup is shown in Figure 1b. The figure shows a custom-designed drilling system ③ along with coordinate frames and corresponding transformations. Here, the notation TBA denotes the transformation from frame {B} to frame {A} . The optical camera ⑥ with frame {C} helps to localize the optical marker ⑧ attached to the spine phantom {spine} and the optical marker ⑦ attached to the drilling system {drill} . These markers are utilized to move the drill tip, expressed with frame {tip} , relative to the robot ① with base frame {R} . Here, the spatial calibration between the drill and the optical camera is performed in advance.

### 2.2 Tool calibration

**FIGURE 2:**
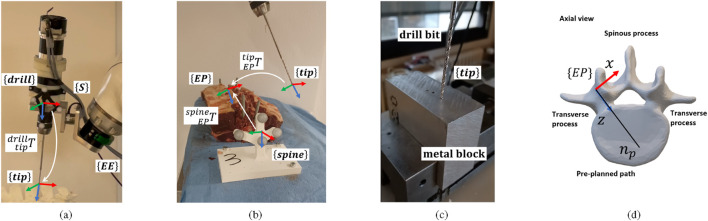
Optical guided drilling system: **(a)** a custom-designed drilling system and its corresponding frames. **(b)** An experimental phantom and its corresponding frames. **(c)** The metal block is used for calibration. **(d)** Entry point definition for each pedicle.

To achieve good accuracy, a careful calibration of the optical guided drilling system is essential. Here, the calibration aims to identify the homogeneous transformation Ttipdrill between the tip frame {tip} and the marker frame {drill} . Figure 2a shows the drill optical marker where rigidly attached to the drilling system, and its frame definition is similar to the tip frame. In this work, a dedicated calibration procedure on the assembled and mounted drilling system is conducted to identify this transformation. Figure 2 shows the drilling system and a calibration setup to determine the actual transformation matrix Ttipdrill . The calibration procedure for position and orientation errors is performed separately.

#### 2.2.1 Drill tip position calibration

**FIGURE 3:**
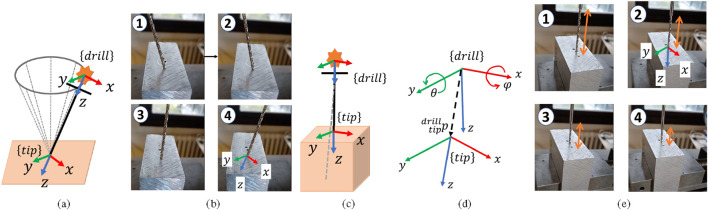
Position calibration setup: **(a)** drill tip pivoting. **(b)** Example of pivoting the drill tip in practice. Orientation calibration setup: **(c)** linear movement along drill axis (gray-dash line). **(d)** Drilling frame and the tip frame. **(e)** Example of linear movement of the drill tip in practice.

A pivot point calibration procedure is performed to estimate ptipdrill , the position of the drill tip relative to the drill marker {drill} Birkfellner et al. (1998). During this calibration, the drill tip is kept in fixed contact with a rigid calibration surface (see Figure 3b) by applying a constant force along its z-axis. While maintaining this fixed pivot point, the robot moves the drilling system within a conical workspace of approximately ±20° , as illustrated in Figure 3a. Several practical factors could affect the calibration quality of the pivoting approach. For example, applying excessive force or moving at high speed may cause the drill bit to dent the surface, to slip or to bend during the pivoting process. Therefore, during the calibration procedure, the robot’s end effector (EE) applies a force of approximately 3N moving at 10mm/s , which is relatively slow for the setup. Additionally, the pivot point is selected to coincide with a small hole on the upper surface of a metal calibration block well within the robot’s workspace, as shown in Figure 3b to ensure that the drill tip does not slip. The low 3N force prevents further deepening the dent in the metal surface. For each configuration i , the corresponding drill marker’s position pidrillC∈R3×1 and orientation RidrillC∈SO(3) are recorded and used to determine the tip position in the drill marker frame. For each measurement i , the position of the tip relative to the camera frame {C} is:

ptipC=pidrillC+RidrillCptipdrill. (1)

Equation 1 contains the unknown but constant vector ptipdrill∈R3×1 and the unknown constant contact tip vector ptipC∈R3×1 . The index i∈{1,…,N} represents each measurement, and N is the total number of measurements. During each pivot calibration trial, N=100 pose measurements were uniformly sampled over the conical motion. The following cost function can then be minimized to identify the two unknown position vectors:

J=∑i=1NptipC−pidrillC+RidrillCptipdrill2. (2)

By collecting the unknown variables in vector x=[ptipdrill,ptipC]|6×1 , and defining matrices Fi=[RidrillC,I3]|3×6 and bi=[pidrillC]|3×1 , the cost function can be written as:

$$J={\left(\tilde{F}x-\tilde{b}\right)}^{T}\left(\tilde{F}x-\tilde{b}\right),$$ (3)

with,

$$\begin{array}{cc} \tilde{F} & ={\left[F_{1}^{T},\;F_{2}^{T},\ldots ,F_{N}^{T}\right]}^{T}\;{|}_{3N\times 6}\\ \tilde{b} & ={\left[b_{1}^{T},\;b_{2}^{T},\ldots ,b_{N}^{T}\right]}^{T}\;{|}_{3N\times 1}. \end{array}$$ (4)

Then the unknown variable can be found by least square optimization:

$$x={\left({\tilde{F}}^{T}\tilde{F}\right)}^{-1}{\tilde{F}}^{T}\tilde{b}.$$ (5)

#### 2.2.2 Drill tip orientation calibration

A calibration procedure is performed to estimate Rtipdrill , the orientation matrix of the drill tip relative to the drill marker {drill} . For calibrating the orientation, a hollow cylinder with an inner diameter of 3.6 mm was used. Given the 3.5 mm outer diameter of the drill, it is possible to slide the drill into the cylinder phantom. This movement constrains the motion to a straight path. The position of the drill tip is measured in the marker frame {drill} , illustrated by the dashed gray line in Figure 3c. Assume that the dashed black line (in Figure 3d) represents a vector originating from the measured tip frame within the drill marker frame, denoted as ptipdrill . If the tip frame {tip} is not perfectly aligned with the drill marker frame {drill} (see Figure 3d), this measured line is not parallel to the z-axis of the drill marker frame {drill} , and it has a projection in the xy-plane of the drill marker frame {drill} . Here, the orientation errors are the rotations around the x and y axes of drill marker frame {drill} , which can be defined as φ and θ rotation angles, respectively. The tip points, pjtipdrill for j∈1,…,M , are measured with respect to the initial drill marker frame {drill} . During orientation calibration as shown in Figure 3e, the drill is translated along a 50 mm linear stroke, and M=100 tip samples pjtipdrill are recorded in the drill marker frame. The Singular Value Decomposition (SVD) method is then applied to determine the direction of these points Muller et al. (2004). The unit vector corresponding to the highest singular value provides the direction vector that is the z-axis of the tip frame {tip} . This direction is represented by the unit vector $\tilde{v}=\left[v_{x},v_{y},v_{z}\right]$ , leading to:

$$\tilde{v}={\left[sin\left(\theta \right),-sin\left(\varphi \right)cos\left(\theta \right),\;cos\left(\varphi \right)cos\left(\theta \right)\right]}^{T}.$$ (6)

Then, the orientation error can be extracted from:

$$\left\{\begin{array}{l} \varphi =\text{atan}\left(-v_{y},v_{z}\right)\\ \theta =\text{atan}\left(v_{x},\sqrt{v_{y}^{2}+v_{z}^{2}}\right) \end{array}\right..$$ (7)

Since rotation around the z-axis is not important for the drill system, this rotation angle ψ is set to zero. Knowing the rotation angles $\left[\varphi \right.$ , θ , $\left.\psi \right]$ helps to regenerate the calibration orientation matrix Rtipdrill by using Euler angles with X-Y-Z successive rotation angles. Then, the calibration matrix can be summarized as:

Ttipdrill=Rtipdrillptipdrill01×31. (8)

If the marker on the drilling system is moved or the drill bit is exchanged or wears off, for good precision, the calibration procedure must be repeated prior to drilling.

### 2.3 Pedicle entry point tracking

The optical camera then helps to localize the relative position of the entry point frame {EP} and the drill tip frame {tip} in real-time, denoted as TEPtip (see Figure 2a). For each entry point frame {EP} , the z-axis is along the pre-planned drilling path $n_{p}$ , the x-axis points towards the spine middle plane, and the y-axis is calculated accordingly, as shown in Figure 2d. To track {EP} , both the drill marker {drill} and the spine phantom marker {spine} (as shown in Figure 1b) are measured in the camera frame {C} . The relative motion TEPtip that is needed to align the drill tip with the preoperatively defined entry point {EP} can then be found as:

TEPtip=T−1tipdrill  T−1drillC  TspineC   TEPspine. (9)

where TEPspine is the defined entry point frame on the vertebral bone surface in the spine marker frame (see Figure 2a), and Ttipdrill denotes the tip calibration in the drill marker frame. While the drill tip approaches the entry point, the frames eventually overlap, and TEPtip will thus converge to I . At that instant in time, the drill tip is aligned with the preoperative trajectory. However, to control the robot to this end, the desired motion must be represented in the robot base frame. The desired trajectory to track the entry point can then be calculated as follows:

TEPRk=TtipRkTEPtipk. (10)

where TtipR(k) is the transformation from the robot base to the tip at time step k . It is decomposed into the desired tip pose ${\text{P}}_{d}=\left[\tilde{p},\tilde{\theta }\right]$ , where $\tilde{p}\in R^{3\times 1}$ and $\tilde{\theta }\in R^{3\times 1}$ are the position and Euler orientation angles of the tip frame with respect to the robot base frame.

### 2.4 Robot control

**FIGURE 4:**
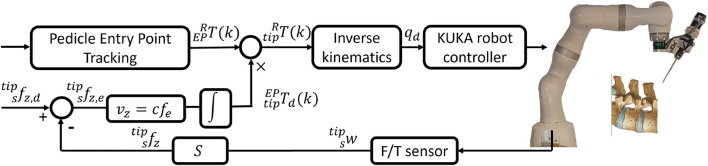
The schema of proposed hybrid control of drilling system.

After reaching the desired entry point and desired orientation, summarized as TEPR(k) , the robot drills into the bone along the planned trajectory. A hybrid position/force control is implemented to maintain the drill tip’s alignment while exerting a constant force along the drilling direction. Figure 4 shows the control scheme to realize this behavior. A six-axis force/torque (F/T) sensor is rigidly mounted between the robot flange and the drilling tool, as shown in Figure 2a. The sensor measures the interaction wrench $sw={\left[f_{s},\tau_{s}\right]}^{T}\in R^{6}$ , expressed in the sensor frame {s} . To obtain the wrench at the drill tip and express it in the drill-tip frame {tip} , the measured wrench is transformed using the spatial wrench transformation:

wtip=X*stipws, (11)

where X*stip denotes the dual adjoint transformation matrix constructed from the known rigid-body transformation Tstip between the sensor frame {s} and the drill-tip frame {tip} . The dual adjoint matrix is given by:

X*stip=Rstip0p×stipRstipRstip, (12)

where Rstip∈SO(3) is the rotation matrix and pstip∈R3 is the translation vector from {s} to {tip} . The matrix p×stip denotes the skew-symmetric matrix associated with pstip . This transformation accounts for both the rotational alignment of the frames and the moment contribution caused by the lever arm between the sensor origin and the drill tip. To minimise the influence of measurement noise, a low-noise six-axis force/torque sensor is employed. Therefore, no additional filtering is required. Static bias and tool weight are compensated through an initial zeroing procedure performed prior to drilling, ensuring that only interaction forces at the drill tip are regulated. A selection matrix S=diag([0,0,1,0,0,0]) is used to extract the measured force along the z-axis, ftip=S wtip , from the measured wrench wtip∈R6 . The drilling axis is force-controlled to ensure precise guidance of the drill bit, while position control is applied to the other degrees of freedom (DoFs). To prevent unwanted movement due to the drilling interaction force and to ensure safety (e.g., avoiding damage to critical structures like the spinal cord in case of a breach), an admittance law converts the force error, fetip , into a corresponding velocity in the z-direction:

vztip=cfetip=cfdtip−ftip. (13)

Here, vztip , c , and fdtip represent the velocity of the drill tip along the z-axis of the tip frame {tip} , the control gain, and the desired force, respectively. Structural compliance in the tool–sensor–robot structure is handled by the admittance controller, which reduces the commanded velocity when force deviations are detected, thereby preventing excessive advancement of the drill. The applied drill-tip pose is computed as:

TdtipRk=TEPRk TdtipEPk, (14)

TdtipEPk=I3×3pk01×31, (15)

where

pk=0, 0,∫t0tkvztipt dt⊤

Represents the integrated drill velocity along the drill axis (z-axis of the tip frame) from the initial time $t_{0}$ to time $t_{k}$ . Thus, TdtipEP(k) corresponds to the incremental translation generated by the interaction force controller. The desired drill-tip pose TdtipR(k) is mapped to joint space via inverse kinematics:

qdk=IKTdtipRk. (16)

The desired joint positions $q_{d}\left(k\right)$ are transmitted to the robot controller, which operates with an internal joint-level position control loop. The low-level controller computes the required motor torques internally to track the commanded joint positions. Consequently, torque computation is handled by the manufacturer’s embedded controller and is not explicitly implemented in our framework.

### 2.5 Experimental plan

#### 2.5.1 Experimental setup

The experimental setup consists of a lightweight robotic arm (KUKA Robot MED7, Augsburg, Germany) and an OTS (Fusion Track 500, Atracsys, Switzerland), as shown in Figure 1b. A custom-designed drilling system is mounted onto the robot’s end effector using a fast tool changer (G-SHW063-2UE, GRIP GmbH, Germany). An external 6 DoF force/torque sensor (Nano25, ATI Industrial Automation Inc., US) is integrated into the drilling system to measure the interaction force and torque at 200 Hz. A PC workstation (Intel i9, CPU @2.6 GHz, 32 GB RAM) is used for robot control, and the operating system is Ubuntu 20.04.6 LTS. The Linux kernel is patched to operate in real-time, and the controller runs via KUKAs Fast Robotic Interface (FRI, version 1.15) at 200 Hz. The Robot Operating System (ROS2) and Open Robot Control Software (Orocos, version 2.9.0) are the middlewares used to implement real-time robot control.

#### 2.5.2 Experiment design

**FIGURE 5:**
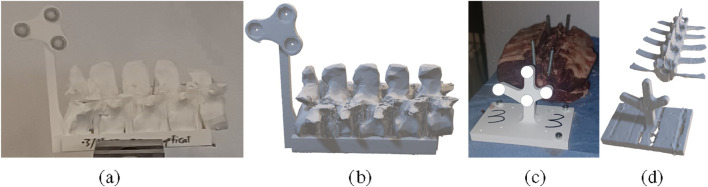
View on the different experimental phantoms with optical markers: **(a)** 3D printed phantom of the lumbar spine, **(b)** CT reconstructed model of 3D printed phantom, **(c)** *ex-vivo* ovine specimen phantom, and **(d)** the corresponding reconstructed CT model.

The experiment is carried out on 3D print phantoms and *ex-vivo* ovine spines visible in Figure 5. A lumbar spine model (L1 to L5) is 3D printed for experimental validation in the laboratory. An optical marker is attached to the 3D printed lumbar model. The model is printed three times to assess the system’s repeatability. Before drilling into each 3D printed phantom, the hard outer layer of the model is removed manually to reduce the slippage of the drill tip on the phantom. The 3D-printed phantom, being lightweight, was rigidly secured using a portable machine bench clamp to prevent displacement caused by drilling interaction force. In this step, approximately 1–2 mm of the surface is removed. In total, 10 screw paths, 5 on each side of the spine phantom, are planned by the operator, resulting in 30 screw paths (3 spines × 5 vertebrae × 2 paths). The paths are planned in the center of the pedicle with maximum clearance from the cortical bone.

Three ovine lumbar spines, each containing 8 vertebrae, are used for the *ex-vivo* validation. Each spine is attached to a wooden plate using screws. The combined weight of the spine and wooden plate provided sufficient stability during robotic drilling without additional fixation to the table. Then, an optical marker is 3D printed and attached to the wooden surface. Subsequently, a preoperative CT scan is performed. The preoperative CT model of the ovine spine is segmented from 2D images then registered to the spine marker frame before trajectory planning. Then, two screw paths are planned per ovine vertebra, resulting in 48 screw paths (3 spines × 8 vertebrae × 2 paths). The drill tip was re-calibrated before each experiment and the calibration matrix was verified to remain consistent across the experiment. There is nothing to report on ethical approval since this research does not contain studies with human participants or living animals, and ovine lumbar spins are bought from the butchery store.

### 2.6 Evaluation criteria

Prior to evaluating the overall drilling system, the calibration accuracy was first investigated independently. Therefore, to evaluate the repeatability of the proposed tool calibration procedure, the complete calibration sequence was repeated 10 times under identical experimental conditions. The drill tip was initially positioned against the calibration surface, and no changes were made to the marker attachment or system configuration between repetitions. For each trial, N=100 pivot poses were recorded within a ±20° conical motion for tip position estimation, and M=100 tip samples were collected over a 50 mm linear stroke for orientation estimation. The pivot fit residual was computed to quantify how well the fixed pivot constraint is satisfied during the least-squares estimation in Equation 2. After estimating ptipdrill and ptipC , the pivot fit residual for each trial was defined as the mean Euclidean distance:

r¯=1N∑i=1NpidrillC+RidrillCptipdrill−ptipC2. (17)

This metric reflects the consistency of the pivot assumption and quantifies the fitting error of the pivot-based position calibration. For the orientation calibration, the tilt angles φ and θ were estimated according to Section 2.2.2. Considering K=10 as number of repeated calibration trials, the mean tilt angles were computed as:

$$\bar{\varphi }=\frac{1}{K}\overset{K}{\underset{k=1}{\sum }}\varphi_{k},\;\bar{\theta }=\frac{1}{K}\overset{K}{\underset{k=1}{\sum }}\theta_{k}.$$ (18)

**FIGURE 6:**
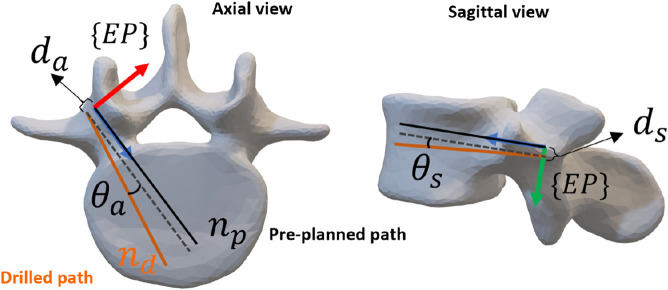
Definition of position and orientation error. The drilled screw path is in orange, the corresponding preoperative plan is in dark.

Two criteria are used to evaluate the functionality of the developed system. The first type consists of geometric errors. Here two vectors ndEP , the drilled path, and npEP , the planned path, are defined. Both are expressed in the entry point frame {EP} . The variables $EPd_{a}$ and $EPd_{s}$ denote the position errors along the axial and sagittal planes, respectively. Similarly, θaEP and θsEP represent the axial and sagittal orientation errors between the drilled and planned paths (see Figure 6). The overall entry point distance error, denoted as dEP , and the overall angle error, denoted as θEP , are then computed:

dEP=daEP2+dsEP2,θEP=cos−1npEP.ndEP|npEP|×|ndEP| (19)

Beside geometry errors, the surgical outcome is also evaluated by classifying pedicle breaches according to the GR grading classification. The GR grading system foresees four different scales: Grade A is assigned when the screw is fully within the pedicle, without cortical breach. Grade B stands for a minor cortical breach where the breach is not higher than 2 mm. Grade C is a moderate cortical breach that is between 2 and 4 mm, whereas Grade D is considered a severe cortical breach for breaches that exceed 4 mm breach (Robbins and Gertzbein, 1989). If the largest cortical breach is less than 2 mm, the corresponding grade is labeled successful with a label of 1. Otherwise, it is labeled 0, indicating that the drilling action was unsuccessful (Burström et al., 2020). Clinical accuracy is then assessed by aggregating the grades of all the drilled screw trajectories. This classification is clinically used during postoperative quality assessment *after* inserting the appropriate screw into the pedicle. The size of the screw that is introduced in the drill hole influences the resulting GR grade. In clinical practice the screw diameter is selected in function of the size of the pedicle. The screw diameter should preferentially fill 80 % of the pedicle’s volume (Solitro et al., 2019). Additionally, bone quality also plays a role in screw selection. If the cancellous layer of the vertebrae is sufficiently dense, smaller screw diameters may be appropriate for PSP.

**FIGURE 7:**
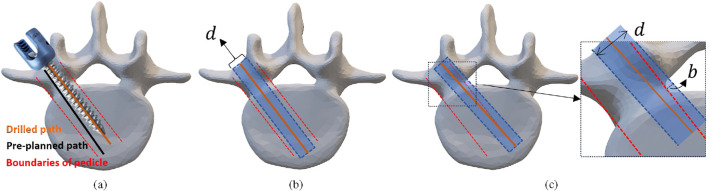
**(a)** Illustration of the Gratzbein grade error definition. **(b)** Accurately placed screw **(c)** screw with a medial breach perforation.

However, in this study, the appropriate screw diameter for each pedicle was determined based on the measured pedicle size. All pedicles were drilled using a 3.5 mm diameter drill bit. Instead of inserting physical pedicle screws and assessing their postoperative position, the screw placement accuracy was evaluated numerically. For a clearer understanding of this assumption, see Figure 7a. Postoperative CT images were used to segment the drilled hole. A cylindrical model was fitted and aligned with the segmented drill trajectory to estimate the executed drilling axis. The cylinder diameter was then expanded to simulate clinically relevant pedicle screw sizes. Specifically, each executed trajectory was extrapolated using cylindrical models with diameters of 4, 5, 6, and 7 mm, corresponding to commonly used pedicle screw dimensions (Burström et al., 2020). Figure 7b illustrates a Grade A placement, in which the simulated cylindrical screw with diameter d (blue area) remains fully within the pedicle cortical boundaries (red contour). In the event of a cortical breach, as shown for a medial perforation in Figure 7c, the simulated screw extends beyond the pedicle boundary, resulting in a breach of size b . According to the magnitude of this breach, the placement is classified as Grade B, C, or D following the GR criteria. This numerical approach allows consistent geometric assessment of screw placement accuracy while avoiding additional mechanical variability associated with physical screw insertion.

## 3 Results

The calibration errors across the 10 repetitions are summarised in Table 1. The mean tip position residual was 0.28±0.17 mm, indicating good stability of the pivot calibration. The orientation calibration showed small angular deviations, with mean tilt angles of 0.85±0.13° for φ and 1.2±0.14° for θ . These results demonstrate that the proposed calibration method provides consistent and repeatable estimation of the drill tip transformation, with sub-millimetre positional variability and sub-degree angular variability. Therefore, tool calibration is unlikely to be a dominant contributor to the overall drilling error.

**TABLE 1:** Residual errors of the tool calibration over 10 repetitions (mean ± SD).

Metric	Mean	SD
Tip position residual (mm)	0.28	0.17
Tilt angle φ (deg)	0.85	0.18
Tilt angle θ (deg)	1.22	0.14

**FIGURE 8:**
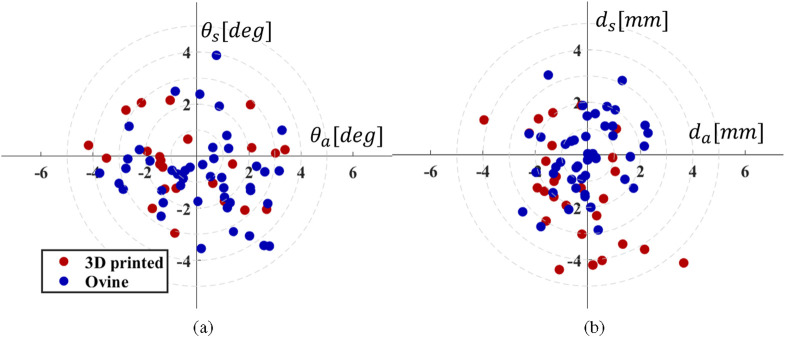
Experimental results: drilling performance expressed as: **(a)** angle deviation and **(b)** position error at the entry point in axial and sagittal planes. The red dots represent the results on 3D printed phantoms, while the blue dots represent the results on *ex-vivo* ovine spines.

A total of 30 screws were drilled into synthetic phantoms and 48 into *ex-vivo* ovine spines. Tables 2, 3 summarizes the obtained experimental results for position and orientation errors. The mean position errors, $d_{a}$ and $d_{s}$ , for the *ex-vivo* ovine spines are −0.07±1.17 mm and −0.06±1.36 mm, respectively. However, due to the skiving of the drill tip despite the preparation and the mesh pattern inside the 3D printed materials, these errors increase to −0.44±1.57 mm and −1.30±1.93 mm in the 3D printed phantoms. The orientation errors $\theta_{a}$ and $\theta_{s}$ on the ovine phantom are 0.19±1.83° and −0.70±1.54° , respectively. This mean non-zero error in position and orientation comes from either the residual calibration error or inaccuracies in the registration of the CT frame to the spine marker frame. Still, the current results on the ovine phantom demonstrate that the calibration routine used is adequate for a robotic PSP system because of its near-zero error. However, the standard deviation in orientation and position shows the errors’ repeatability and directly affects the functional and surgical errors. The individual position and orientation error of the entry points are depicted in the Figure 8.

**TABLE 2:** Results of geometry performance evaluation of drilled screw path on 3D printed spines.

Exp.	$d_{a}$ (mm) mean	$d_{s}$ (mm) mean	$\theta_{s}$ (deg) mean	$\theta_{a}$ (deg) mean	d (mm) median (IQR 25, 75)	θ (deg) median (IQR 25, 75)
1	0.02	−1.16	−0.85	−0.82	2.09 (1.41, 2.98)	2.03 (1.47, 3.49)
2	−0.60	−1.28	0.40	−0.81	2.12 (1.70, 3.60)	2.71 (1.44, 2.94)
3	−0.82	−1.47	−0.60	−0.87	2.33 (1.94, 3.79)	3.08 (1.88, 3.37)
Mean	−0.44	−1.30	−0.39	−0.84	2.16 (1.62, 3.49)	2.65 (1.47, 3.34)
Std	1.57	1.93	2.19	2.20		

**TABLE 3:** Results of geometry performance evaluation of drilled screw path on ovine spines.

Exp.	$d_{a}$ (mm) mean	$d_{s}$ (mm) mean	$\theta_{s}$ (deg) mean	$\theta_{a}$ (deg) mean	d (mm) median (IQR 25, 75)	θ (deg) median (IQR 25, 75)
1	0.12	0.63	−0.23	−0.42	1.48 (0.94, 2.00)	2.34 (1.25, 2.77)
2	−0.69	−1.09	0.69	−0.56	1.69 (1.16, 2.17)	2.45 (2.12, 3.37)
3	−0.31	0.29	−0.08	−1.06	1.39 (0.73, 2.06)	1.82 (1.20, 2.89)
Mean	−0.07	−0.06	0.19	−0.70	1.51 (0.91, 2.10)	2.30 (1.28, 3.13)
Std	1.17	1.36	1.83	1.54		

**FIGURE 9:**
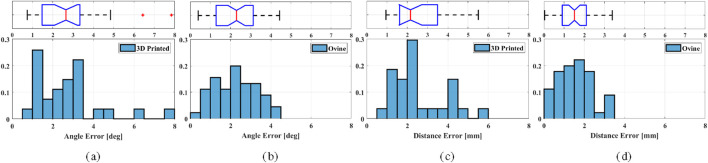
Histogram showing the probability distribution of drilling errors on 3D printed phantoms (n=30) and *ex-vivo* ovine spines (n=48) : angle errors at the entry point for **(a)** 3D printed and **(b)** ovine spines, distance errors at the entry point for **(c)** 3D printed and **(d)** ovine spines.

Figure 9 demonstrates the histogram of the obtained performance for drilling experiments on synthetic phantoms and *ex-vivo* ovine spines. The box plots depict the median as a solid red line, the hinges correspond to the first and third quartiles, and the dots represent outliers. The median distance errors are 2.16 mm (IQR 1.62mm, 3.49 mm) on 3D printed phantoms and 1.51 mm (IQR 0.91mm, 2.10 mm) on *ex-vivo* ovine spines. The maximum error of 75% interquartile distance in the 3D printed phantom is 3.79 mm in experiment 3, while the maximum error on the ovine spine is 2.17 mm. To evaluate whether performance differed between the two experimental conditions, a statistical comparison was performed using Welch’s t-test to account for unequal variances. No statistically significant difference was found for angular error (p=0.138) . However, the entry-point distance error was significantly higher in the 3D-printed group $\left(p=6.57\times 10^{-4}\right)$ . The mean difference (ovine minus 3D-printed) was −0.98 mm with a 95% confidence interval of [−1.52,−0.44] mm, indicating reduced translational accuracy in the synthetic phantom.

**FIGURE 10:**
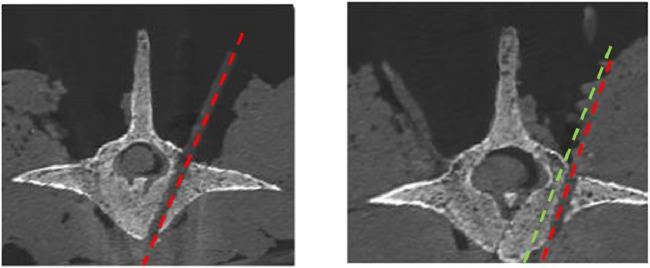
Examples of the drilled screw path on postoperative CT with a case of Grade A and Grade B.

Table 4 shows the GR Grade for four different screw diameters, based on the measured drilled screw path in the *ex-vivo* ovine spines. With a screw diameter of 4 mm, the 48 screws achieve a 100% success rate because 4 happened breaches are within 2 mm. However, the clinical success rate decreases to 97.91% and 93.75% if screws diameter 5 mm and 6 mm were to be inserted, respectively. In total, 19 out of the 48 screws achieve Grade A when using a 7 mm screw. Since ovine spine vertebrae vary in shape and size compared to human spines, the proper screw diameter must be selected based on the 80% fill of the pedicle canal. Selecting the appropriate screw size results in a 95.83% success rate; specifically, screw diameters of 5 mm and 6 mm were found to be the most suitable. Figure 10 shows the drilled screw path (red line) and desired path (green line) on postoperative CT scans for two cases: one with Grade A and one with Grade B. In this example, the Grade B case is found to exhibit a minor lateral breach.

**TABLE 4:** Results of the Gertzbein-Robbin Grade of the drilled screw on the *ex-vivo* ovine spines and the corresponding clinical accuracy. ∗ refers to the proper screw diameter based on the pedicle size.

Screw diameter	NO. of screws				Clinical accuracy [%]
$\left[mm\right]$	A	B	C	D	(A + B)/total
4	44	4	0	0	100
5	41	6	1	0	97.91
6	32	13	3	0	93.75
7	19	23	6	0	87.50
∗	35	11	2	0	95.83

## 4 Discussion

In previous research work by Li et al. and Fu et al., the authors reported superior surgical outcomes for the robot-assisted pedicle screw placement technique compared to the conventional freehand technique in terms of the Gertzbein-Robbins grade and shorter postoperative stay (Fu et al., 2021; Li H.-M. et al., 2020). Conventional commercial robot-assisted systems operate as follows: surgeons preoperatively determine screw trajectories and diameters based on CT scans and match them with intraoperative fluoroscopy for instrumentation (Xie et al., 2023; Hyun et al., 2017). A guide holder mounted on the robot arm automatically moves to the target entry point according to the surgical plan (Yan et al., 2022). However, the surgeon is still required to perform the drilling procedure manually with a poor field of view and spine deformations this may lead to screw misplacement (Vafadar et al., 2023). This work proposes an optical-guided robot-assisted system for PSP. The vertebra’s entry point is tracked for each individual pedicle. After reaching the entry point, a hybrid position/force control is used to drill the pedicle. Although a preoperative CT scan is still required, this method could significantly decrease the radiation dose compared to fluoroscopy-based intraoperative navigation. With respect to radiation exposure, it is important to differentiate between patient dose and occupational exposure to the surgical team (Fang et al., 2005; Jones et al., 2000). Intraoperative 3D imaging (e.g., cone-beam CT or O-arm) primarily increases patient dose, whereas repeated fluoroscopy contributes to scatter radiation affecting surgeons and operating room staff. By relying on optical-guided robotic drilling, the proposed workflow reduces the need for repeated fluoroscopic verification, potentially lowering radiation exposure, while the patient’s radiation dose remains dependent on the chosen imaging protocol. The discussion of this study is divided into three parts:

### 4.1 Elaboration of the obtained result

The developed optical-guided robot-assisted system for PSP was validated with both synthetic phantoms and *ex-vivo* ovine spines. In total, 30 screws were drilled into synthetic phantoms, resulting in an error of 2.16 mm (IQR 1.62mm, 3.49 mm) in distance and an error of 2.65° (IQR 1.47° , 3.34° ) in angle at the entry point level. For 48 screws on *ex-vivo* ovine spines, the distance error was 1.51 mm (IQR 0.91mm, 2.10 mm) while the angle error was 2.30° (IQR 1.28° , 3.13° ). This accumulated error may come from several sources such as: the reported robot repeatability (0.1 mm) or nonlinearity (0.1 mm), measurement errors of the OTS (0.22 mm), but also from remaining tool calibration errors, skiving and bending of the drill or the inherent flexibility and movement of the spine and its vertebrae which is not measured separately in this study. However, the measurements of the error in the drilled screw path are not directly comparable with conventional grading scales. The deviation of over 2 mm in the proposed system does not directly equate to a grade C or a worse rating. A screw placement that is clinically acceptable according to the conventional grading scale can still deviate significantly (Volk et al., 2023). With a 4 mm screw diameter, the clinical accuracy achieved a success rate of 100%. However, as mentioned, the choice of screw diameter depends on the pedicle size and the patient’s bone quality. If the 4 mm screw would establish a decent screw hold (insertional torque) the current performance would be considered clinically acceptable; otherwise, a larger diameter screw should be chosen. Table 4 demonstrates that placing a screw with a larger diameter would, at this stage of development, results in a GR Grade C. Part can be explained because the pedicle area of the ovine spine is smaller than that of a human, resulting in a worse grading scale when the screw diameter is increased. From a clinical perspective, the choice of screw diameter is determined by the surgeon based on the patient’s pedicle morphology and bone quality. In human lumbar vertebrae, pedicle dimensions are generally larger than in the ovine model used here, which would provide greater cortical clearance for the same drilling accuracy. Therefore, the clinical success rate of the proposed system is expected to improve when applied to human anatomy.

### 4.2 Comparison to the state-of-the-art

A structured comparison with existing robotic and navigation-assisted spinal systems is presented in Table 5. Several robotic systems rely on fluoroscopic guidance or intraoperative CT imaging. Gao et al. developed a fluoroscopy-guided robotic needle injection system, reporting a mean translational error of 5.1±2.4 mm and orientation error of 3.6±1.9° in cadaver studies (Gao et al., 2022). While demonstrating feasibility, their approach differs in both clinical objective (needle injection rather than pedicle drilling) and imaging modality (radiation-based fluoroscopy). Commercial robotic systems, such as the one evaluated by Vardiman et al., achieved a 97.7% Grade A + B success rate in clinical practice; however, these systems rely on intraoperative CT-based navigation and provide only a robotic drill guide for PSP (Vardiman et al., 2020). CT-guided robotic pedicle screw placement has also been investigated by Kisinde et al., who reported an overall deviation of 2.20±1.17 mm for cervical screws with a 15.9% minor medial breach rate ( ≤ 1 mm) (Kisinde et al., 2022). Their work was performed in clinical cervical cases using robotic guidance integrated with CT imaging, whereas our study focuses on thoracolumbar trajectories in an *ex-vivo* ovine model with optical tracking and postoperative quantitative trajectory analysis. Radiation-free alternatives have been explored using ultrasound-based navigation. Li et al. proposed a robot-assisted ultrasound-guided system for minimally invasive pedicle screw placement and reported an entry point error of 2.39±1.41 mm and orientation error of 2.82±1.85° with an 87.5% GR success rate in ovine models (Li et al., 2024). Similarly, Gueziri et al. demonstrated intraoperative ultrasound navigation with a 92.86% success rate in porcine lumbar spines, although drilling was performed manually (Gueziri et al., 2022). Vafadar et al. proposed force-controlled robotic drilling with probing-based registration and achieved a mean orientation error of 2.2° and distance error of 2.2 mm in porcine lumbar vertebrae (Vafadar et al., 2023). Beyond spine surgery, Bakhtiarinejad et al. reported a distance error of 3.28 mm and orientation error of 2.30° in robotic hip augmentation using fiducial-less 2D/3D registration, highlighting the performance of image-based robotic drilling in a different anatomical context (Bakhtiarinejad et al., 2023). Compared to these approaches, the present work achieves a median entry point error of 1.51 mm and angle error of 2.30° with a 95.83% GR clinical success rate. Importantly, our framework combines optical navigation with autonomous robotic drilling and quantitative postoperative validation, providing competitive accuracy while avoiding continuous intraoperative fluoroscopy.

**TABLE 5:** Comparison with state-of-the-art robotic and navigation-assisted spinal systems.

Study	Imaging modality	Model	Task	Distance error (mm)	Angle error (°)	Success rate
Bakhtiarinejad et al.	2D/3D image-based	Cadaver	Hip drilling/injection	3.28	2.30	–
Gao et al.	Fluoroscopy	Cadaver	Needle injection	5.1±2.4	3.6±1.9	–
Kisinde et al.	CT-guided	Clinical	Cervical PSP	2.20±1.17	–	84.1%
Vardiman et al.	CT-guided (commercial)	Clinical	PSP	–	–	97.7%
Li et al.	Ultrasound	Ovine/Cadaver	PSP	2.39±1.41	2.82±1.85	87.5%
Gueziri et al.	Ultrasound	Porcine	PSP (manual)	–	–	92.86%
Vafadar et al.	Probing-based	Porcine	Robotic drilling	2.2	2.2	–
This work	Optical navigation	Ovine (*ex-vivo*)	Robotic drilling	1.51	2.30	95.83%

### 4.3 Limitations of the study

The results obtained from the in-lab experiment do not account for some clinical factors. For example, the pedicle canal of an ovine is smaller than that of a human, leading to a lower success rate for drilling. Additionally, the bone properties of ovines differ from those of humans. To bridge the gap to pre-clinical practice, *ex-vivo* human cadaver and *in vivo* animal experiments should be conducted. Future studies should investigate the system’s performance in dynamic surgical scenarios involving respiration-induced motion, as motion compensation was not assessed in the present study. Potential respiratory motion compensation approaches include optical tracking of a dynamic reference frame attached to the vertebra for real-time pose updates Liu et al. (2016), respiratory phase gating (e.g., end-expiration synchronisation) Dürichen et al. (2013), and predictive motion modelling techniques such as Kalman filtering or deep learning-based estimation methods, which are compatible with standard operating room workflows (Li B. et al., 2023; Davoodi et al., 2024). Further, the accuracy of optical navigation methods relies on the visibility of the optical marker and where it is attached to the spine. In open surgical procedures, soft tissue and surgical instruments may intermittently occlude the optical markers, potentially interrupting tracking continuity. While the current *ex-vivo* setup provides unobstructed line-of-sight, clinical deployment would require redundant marker configurations to ensure robust tracking. Vertebrae farther separated from the optical marker are expected to show larger orientation and position errors due to spinal flexibility. In the current setup, the spine phantom is rigidly fixed to a wooden board, effectively minimizing flexibility. In real clinical scenarios, patients are typically stabilized on the surgical table to reduce motion, but spinal deformation is not completely eliminated. In multi-level clinical procedures, the cumulative effect of spinal flexibility across several vertebral levels may amplify registration errors for vertebrae distant from the reference marker. Strategies such as re-registration at each vertebral level or the use of multiple reference markers should be investigated. By using high-speed drilling at least the interaction force and its effect can be somewhat reduced.

## 5 Conclusion

This study demonstrates that optical navigation alone combined with robotic drilling shows promise for PSP in spine surgery. The findings indicate that such a system could offer a viable alternative to conventional fluoroscopy and CT navigation techniques. The research introduces a robot-assisted drilling system specifically designed for spine surgery. It utilizes optical markers and preoperative CT trajectory planning to drill synthetic phantoms and *ex-vivo* ovine spines. By integrating optical tracking into robotic systems, the accuracy of surgeries is enhanced through real-time detection of patient positioning. Although there are still challenges to address, robots equipped with optical tracking show considerable potential for clinical use. The progress in robotic and navigational technologies outlined in this study suggests a future of more precise, efficient, and safer surgical interventions with reduced reliance on radiation.

## Data Availability

The raw data supporting the conclusions of this article will be made available by the authors, without undue reservation.
